# Commonly used bowel preparations have significant and different effects upon cell proliferation in the colon: a pilot study

**DOI:** 10.1186/1471-230X-8-54

**Published:** 2008-11-14

**Authors:** Lisa J Croucher, Jonathan P Bury, Elizabeth A Williams, Stuart A Riley, Bernard M Corfe

**Affiliations:** 1 Human Nutrition Unit, School of Medicine and Biomedical Sciences, University of Sheffield, Sheffield, UK; 2 Academic Unit of Pathology, School of Medicine and Biomedical Sciences, University of Sheffield, Sheffield, UK; 3 Department of Gastroenterology, Northern General Hospital, Sheffield, UK

## Abstract

**Background:**

Markers of crypt cell proliferation are frequently employed in studies of the impact of genetic and exogenous factors on human colonic physiology. Human studies often rely on the assessment of tissue acquired at endoscopy. Modulation of cell proliferation by bowel preparation with oral laxatives may confound the findings of such studies, but there is little data on the impact of commonly used bowel preparations on markers of cell proliferation.

**Methods:**

Crypt length, crypt cellularity and crypt cell proliferation were assessed in biopsies acquired after preparation with either Klean-Prep or Picolax. Crypt cell proliferation was assessed by whole-mount mitotic figure count, and by two different immunohistochemical (IHC) labelling methods (Ki-67 and pHH3). Subsequent biopsies were obtained from the same patients without bowel preparation and similarly assessed. Parameters were compared between groups using analysis of variance and paired t-tests.

**Results:**

There were significant differences in labelling indices (LI) between biopsies taken after Klean-prep and those taken after Picolax preparation, for both Ki67 (p = 0.019) and pHH3 (p = 0.017). A similar trend was seen for whole-mount mitotic figure counts. Suppression or elevation of proliferation parameters by bowel preparation may mask any effect due to an intervention or disease.

**Conclusion:**

Commonly used bowel preparations may have significant and different effects on crypt cell proliferation. This should be taken into account when designing studies and when considering the findings of existing studies.

## Background

Oral laxatives are routinely used to cleanse the colon prior to colonoscopy, to permit clear visualisation of the colonic mucosa and minimise the risk of infection. Hyperosmotic preparations such as sodium phosphate (NaP) and Picolax^® ^actively draw water into the bowel lumen, increasing stool bulk and stimulating gut motility[1]. The effect is lavage with rapid transit and evacuation of bowel contents. The fluid and electrolyte imbalance that may occur with preparations of this type precludes their use in patients with renal or congestive heart failure, but they are otherwise well tolerated[2]. Klean-Prep^® ^is principally a lavage solution with some osmotic activity, the large ingestion volume being retained within the colon by polyethelene glycol (PEG), itself relatively inert and poorly absorbed. Although the risk of significant fluid or electrolytes shifts is reduced with PEG based preparations[3], the large ingestion volume can be associated with poor patient compliance[4].

Ideally, a bowel preparation agent should have minimal impact on the colonic mucosa. However, endoscopic and histological abnormalities have been reported, particularly with NaP, and include inflammation, haemorrhage, focal active colitis (FAC), erosion of the surface epithelium and apthoid ulceration [5-7]. One study reported apthoid lesions in 24.5% of patients prepared with NaP, compared to only 2.3% of patients prepared with PEG and concluded that NaP should not be used to prepare patients with suspected inflammatory bowel disease[8]. Another study attributing FAC and aphthoid ulcers to NaP found no lesions in a subset of patients subsequently re-examined without preparation[9]. Sodium picosulfate, the active constituent of Picolax, has been shown to increase the expression of acidic mucin and cytokeratin AE1 in rat colonocytes[10]. The effects of PEG on the morphology of the colonic mucosa are less clear. No significant changes were attributed to PEG in two studies of human and rat colon[6,7]. However, subtle structural changes including mucin depletion, epithelial cell loss and inflammatory cell infiltration of the mucosa have been reported elsewhere[5].

Cell proliferation is a commonly used end-point in studies of the effects of exogenous and genetic factors on colonic mucosal function and cancer risk. However, data on the impact of bowel preparation agents on cell proliferation is sparse. A 136% increase in colonocyte Ki-67 labelling index has been reported in patients prepared with NaP, when compared to the same patients, unprepared[9]. Sodium picosulfate was found in an animal study to have no effect on colonocyte proliferation[11], whilst another detected increased proliferation in response to bisocodyl and sennosides, laxatives with a similar action[12].

To date, there are no published studies examining or comparing the direct effects of sodium picosulfate and PEG on colonocyte proliferation. This study aims to compare the effects of these two commonly used bowel preparative agents on immunohistochemical and whole-mount derived indices of cell proliferation.

## Methods

### Ethical approval and patient groups

Patients attending endoscopy clinics at the Northern General Hospital, Sheffield for an initial colonoscopy were recruited to the study. Patients were all male, over 40 and had normal bowel health. Smokers and diabetics were excluded. Patients prepared for endoscopy with Klean-Prep (n = 4) or Picolax (n = 3) were invited to return four weeks later for sigmoidoscopy without bowel preparation. Two biopsies were taken at the recto-sigmoid junction. This study was approved by the North Sheffield Research Ethics Committee (Reference number: 06/Q2308/93). Informed consent was obtained from all patients.

### Measurement of crypt cell proliferation

The range of markers currently utilised for the assessment of colonocyte proliferation is wide, and may in part account for the variability seen between studies. In this study we employed two recognised immunohistochemical markers of cell proliferation, Ki-67 and pHH3. Ki-67 is expressed throughout the cell cycle, whilst pHH3 (phosphorylated histone H3) is detectable in increasing quantity during the progression from from interphase to prophase. This correlates with a progression from granular to diffuse immunohistochemical staining patterns[13]. Counts for both staining patterns were recorded. Where possible, an additional biopsy from each site was taken for whole-crypt mitotic count, assessed using the microdissection method described by Goodlad, et. al[14].

### Immunohistochemical Analyses

Biopsies were fixed for 24 hours in formalin before paraffin embedding and cutting of serial 4-micron sections at 40-micron intervals. Endogenous peroxidise activity was blocked with 2% hydrogen peroxide. Heat-induced eiptope retrieval (HIER) was performed using a sodium citrate buffer (pH 6.0) for Ki-67 and EDTA (pH 8.0) for pHH3. Non-specific immunoglobulin binding sites were blocked with normal horse serum. Slides were incubated for 1 hour with primary antibodies to Ki-67 (Vector Laboratories VP-K 452) and pHH3 (Upstate 06–570) at 1:100 and 1:200, respectively. Staining was visualised with the Vectastain Universal Elite kit and DAB peroxidise substrate (Vector Laboratories). Sections were counter-stained with Gill's haematoxylin.

A maximum of 10 well-orientated hemi-crypts per biopsy, showing the entire length of the crypt wall from the base abutting the muscularis mucosa through to the junction with the surface epithelium, were included. Images were captured at 20× magnification with a Nikon D5-M camera at 2560 × 1920 resolution, stored without compression and analysed using Nikon NIS-Elements D (v 2.30) software. All cell counts and measurements were made by one observer blind to the status of the biopsy. A subset of scores was confirmed by a second independent observer.

The number of cells showing positive nuclear staining for each antigen was recorded as a fraction of the total cell count per hemi-crypt (labelling index:LI). For pHH3, the LIs for granular and diffuse staining were recorded separately. Crypt lengths for up to five crypts per biopsy were determined.

### Whole mount analysis

Whole biopsies were fixed in Carnoy's fluid, then stored in 70% ethanol. Rehydration through 50% and 25% alcohols and PBS was followed by hydrolysis in 5 M NaOH at room temperature. After staining with Schiff's reagent for 90 minutes, the biopsies were transferred to 45% acetic acid for dissection. Small groups of crypts were isolated from surrounding tissue under a dissecting microscope, then transferred to slides and squashed with thumb pressure under a cover-slip with aqueous mountant. Mitoses were counted at ×40 in up to ten whole crypts per biopsy, through the full crypt depth. Nuclei in all phases of mitosis were counted and expressed as the number of mitoses per crypt.

### Statistics

Statistical analysis was carried out with SPSS (version 11.0). The distribution of values for each parameter was tested for normality using the Kolmorogov-Smirnov test. Indices for paired biopsies (i.e those from the same patient, with and without preparation) were compared using the paired Student t-test. One-way ANOVA was used to test for the equality of the means for each parameter between each group (no preparation, Klean-Prep and Picolax), and the unpaired t-test was used to test for differences between biopsies taken after Klean-prep and those taken after Picolax.

## Results and discussion

### Proliferation indices

Immunohistochemical methods for the assessment of cell proliferation in small biopsies suffer from a number of potential disadvantages. Firstly, it can be difficult to obtain high yields of assessable crypts in histological sections. Secondly, there is concern over the impact of the so called "denominator effect" on the LI. Counting mitotic figures in whole-mount preparations avoids these issues, but does suffer the disadvantage that a second biopsy must be obtained if conventional histolological examination (or additional immunohistochemical staining, e.g. for apoptosis) is required. We compared the statistical reliability of both approaches using the Cronbach's alpha statistic -a measure of between and within-case variability, ranging from 0 to 1 with increasing reliability. The scores were 0.9476 and 0.9164 for Ki-67 and whole mount mitotic counts, respectively, indicating that robust measurements can be obtained without exhaustive sampling of crypts. Moreover, scores from both methods correlated well (Pearson correlation coefficient 0.655, p = 0.008). There is also concern that individual antibody targets may not display expression that is truly restricted to proliferating cells, and therefore give potentially spurious results. It is reassuring that the immunohistochemical markers used in this study both showed similar results.

**Figure 1:**
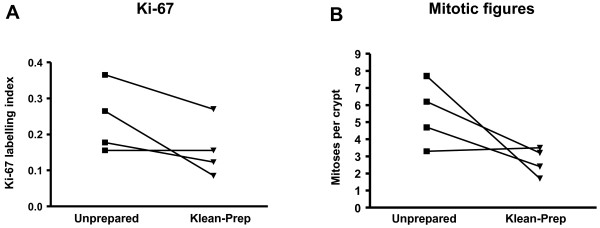
**Paired t-test analyses of proliferative indices in patients prepared with Klean-Prep**. A consistent trend towards suppression of both Ki-67 (A) and mitoses (B) is seen with Klean-Prep compared to unprepared bowel from the same patients.

Two immunohistochemical markers of cell proliferation, Ki-67 and pHH3, were measured. Colonocyte Ki-67 LI decreased after preparation with Klean-Prep, when compared with unprepared bowel from the same patients (Figure 1A). Despite the consistent change, significance was not achieved (p = 0.117, paired t-test). A decrease was also seen in the number of whole-crypt mitoses with Klean-Prep (Figure 1B). Again, the trend was consistent but significance was not achieved (p = 0.118). Klean-Prep had no apparent effect on pHH3 diffuse or granular staining, when compared to that seen in the same patients without preparation (p = 0.519 and p = 0.562, respectively, data not shown).

**Figure 2:**
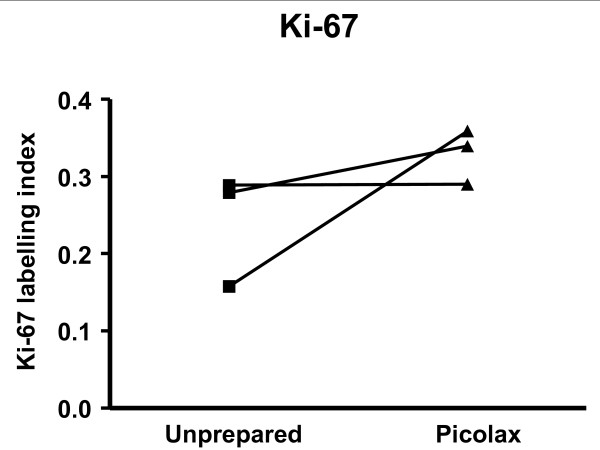
**Paired t-test analysis of Ki-67 LI in patients prepared with Picolax**. A trend towards elevation of Ki-67 LI is seen with Picolax compared to unprepared bowel from the same patients.

Picolax had an opposing effect on colonocyte proliferation. Ki-67 LI increased after preparation with Picolax, although this effect was not significant (p = 0.2778, Figure 2). Similarly, increased diffuse and granular pHH3 staining was observed with Picolax and reached significance in the granular fraction (p = 0.049, p = 0.269 for positive staining, data not shown). Paired samples from only one patient were available for whole crypt staining; the mean number of mitoses per crypt for this patient were 11.4 and 5.0 for Picolax and unprepared biopsies, respectively.

**Figure 3:**
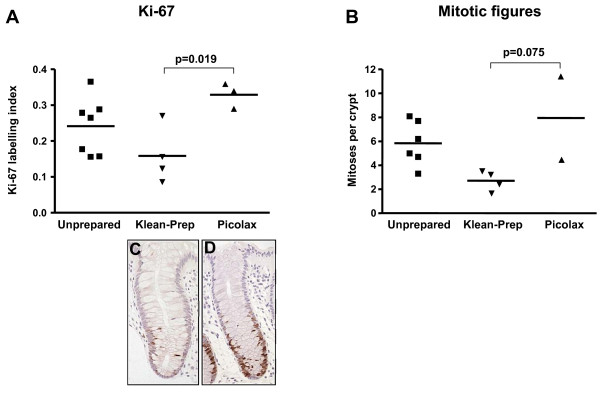
**ANOVA of Ki-67 and mitotic figures in unprepared and prepared bowel**. Significant differences were seen between all groups (p = 0.035 and p = 0.045 for Ki-67 (A) and mitotic figures (B), respectively), with a marked difference in Ki-67 between Klean-Prep and Picolax (p = 0.019). Crypts with a low Ki-67 LI, from a bowel prepared with Klean-Prep, and with a high LI, from a Picolax prepared bowel, are shown in B and C, respectively.

**Figure 4:**
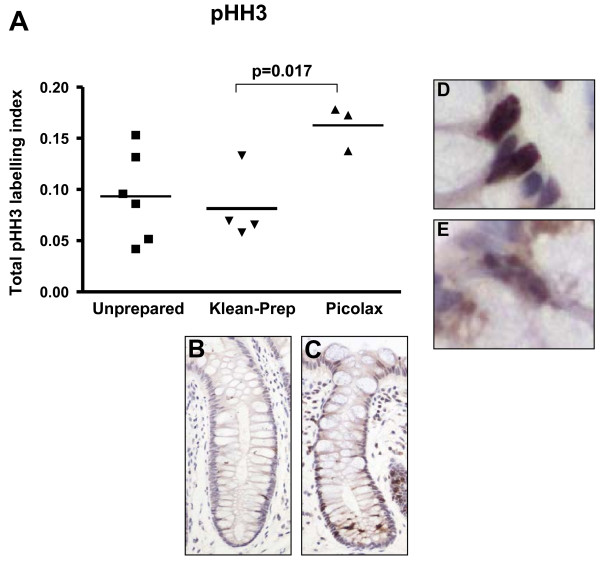
**ANOVA of total (positive and granular) pHH3 in unprepared and prepared bowel**. Significant differences were seen between all groups (p = 0.038), with a marked difference between Klean-Prep and Picolax (p = 0.017). Crypts with a low total pHH3 LI, from a bowel prepared with Klean-Prep, and with a high LI, from a Picolax prepared bowel, are shown in B and C, respectively, as well as examples of positive (D) and granular (E) staining.

While obvious and consistent trends are seen in the above analysis, treating the samples as paired for statistical purposes is undermined by the small numbers (n = 4 for Klean-Prep, n = 3 for Picolax) and the unavailability of paired biopsies for all markers tested. All data, paired or otherwise, was therefore redistributed into treatment groups and subjected to analysis of variance (ANOVA) testing. One-way ANOVA revealed significant differences between groups (Ki-67: p = 0.035, mitoses: p = 0.045), demonstrating, as for the paired analysis, a decrease in proliferation with Klean-Prep, and an increase with Picolax, when compared to unprepared bowel (Figure 3). Scoring differentially for granular and positive pHH3 staining showed a similar pattern of response to bowel preparation (data not shown), but statistical significance was reached when the data sets were combined to give a total LI (p = 0.038, Figure 4). Post-hoc analysis revealed a significant difference between Klean-Prep and Picolax on Ki-67 and total pHH3 LI (p = 0.019 and 0.017, respectively).

This observation may be relevant to the interpretation of proliferation data in several recently published studies. In one study demonstrating no significant difference in Ki-67 or whole crypt mitoses between patients with Hereditary non-polyposis colorectal cancer and normal subjects, the authors concluded that crypt cell proliferation is not a suitable discriminative marker for this disease[15]. All patients had been administered Picolax and, as in our study, had Ki-67 LIs in the range 0.3 to 0.4[16]. A study on the effect of pre-biotic carbohydrates similarly showed no difference in Ki-67 LI between subjects and controls, reporting indices between 0.3 and 0.4. Again, all subjects received Picolax. Our evidence suggests that any marginal change in LI in response to disease or intervention in these studies may have been masked by the proliferative effects of Picolax. Equally, data from studies based on subjects prepared with PEG should be interpreted cautiously. No association was found between the PCNA proliferative index (PI) and the likelihood of developing adenoma in one prospective study; the majority of these patients were prepared with PEG[17]. It is possible that PEG suppressed colonocyte proliferation in a group of patients that might be expected to have a higher than normal PI.

### Crypt length and cellularity

No significant differences were seen by t-test analysis between pairs for either Klean-Prep or Picolax (p = 0.766 and 0.183, respectively), or between groups by one-way ANOVA (p = 0.437). Similarly, there was no significant difference in crypt length between pairs (p = 0.209 for Klean-prep, p = 0.827 for Picolax), or between groups by one-way ANOVA (p = 0.0786) (data not shown). The cellular homeostasis observed despite increased proliferation could be attributed to two factors. Firstly, the interval between bowel preparation and biopsy (typically 12 hours) may have been insufficient for any cells entering cell division to complete the cycle. Secondly, the observed homeostasis may be attributable to increased apoptosis. Our assessment of apoptotic indices recorded very low rates of background apoptosis in all samples, and showed no alteration in response to bowel preparation (data not shown).

### Limitations of study

A randomisation bias on clinical grounds cannot be ruled out in the allocation of patients to Klean-Prep or Picolax preparation; this, along with the small sample size, may in part account for the lack of statistical significance seen between prepared patients and unprepared controls. Similarly, whilst patients prepared with Klean-Prep or Picolax fasted prior to colonoscopy, control patients did not, and potential confounding influences of luminal nutrition and luminal workload on proliferation indices should be noted. However, our analyses, and the central finding of this study, show an opposing direction of change between Klean-Prep and Picolax that is consistent for all proliferation measures, and cannot be a feature of starvation. Although the small number of cases and the single sampling site inevitably means that the results should be interpreted with caution, this pilot study has informed our choice of procedural homogeneity for a separate, larger study requiring repeat endoscopies.

## Conclusion

This study demonstrates that Picolax and Klean-Prep have significantly different effects on crypt cell proliferation. Suppression or elevation of proliferation parameters by bowel preparation may mask any effect due to an intervention or disease.

These data underscore the importance of ensuring that all patients participating in studies in which crypt proliferation is an outcome measure are treated with identical bowel preparation. Bowel preparations used in such studies should be reported, and the data interpreted with caution.

## Competing interests

The authors declare that they have no competing interests.

## Authors' contributions

LJC carried out the analyses and was responsible for drafting the manuscript. JPB contributed to the analyses, performed the statistical analysis and helped to draft the manuscript. EAW participated in the design of the study and assisted with patient recruitment. SR was responsible for patient recruitment and collected biopsy material for the study. BMC conceived the study, participated in its design and helped to draft the manuscript. All authors read and approved the final manuscript.

## Pre-publication history

The pre-publication history for this paper can be accessed here:


